# An artificial intelligence-based risk prediction model of myocardial infarction

**DOI:** 10.1186/s12859-022-04761-4

**Published:** 2022-06-07

**Authors:** Ran Liu, Miye Wang, Tao Zheng, Rui Zhang, Nan Li, Zhongxiu Chen, Hongmei Yan, Qingke Shi

**Affiliations:** 1 MOE Key Lab for Neuroinformation, School of Life Science and Technology, University of Electronic Science and Technology of China, Chengdu, 610054 Sichuan China; 2 Engineering Research Center of Medical Information Technology, Ministry of Education, West China Hospital of Sichuan University, Chengdu, 610041 Sichuan China; 3 Department of Cardiology, West China Hospital of Sichuan University, Chengdu, 610041 Sichuan China

**Keywords:** Artificial intelligence, Myocardial infarction, Machine learning, Imbalanced data

## Abstract

**Background:**

Myocardial infarction can lead to malignant arrhythmia, heart failure, and sudden death. Clinical studies have shown that early identification of and timely intervention for acute MI can significantly reduce mortality. The traditional MI risk assessment models are subjective, and the data that go into them are difficult to obtain. Generally, the assessment is only conducted among high-risk patient groups.

**Objective:**

To construct an artificial intelligence–based risk prediction model of myocardial infarction (MI) for continuous and active monitoring of inpatients, especially those in noncardiovascular departments, and early warning of MI.

**Methods:**

The imbalanced data contain 59 features, which were constructed into a specific dataset through proportional division, upsampling, downsampling, easy ensemble, and w-easy ensemble. Then, the dataset was traversed using supervised machine learning, with recursive feature elimination as the top-layer algorithm and random forest, gradient boosting decision tree (GBDT), logistic regression, and support vector machine as the bottom-layer algorithms, to select the best model out of many through a variety of evaluation indices.

**Results:**

GBDT was the best bottom-layer algorithm, and downsampling was the best dataset construction method. In the validation set, the F1 score and accuracy of the 24-feature downsampling GBDT model were both 0.84. In the test set, the F1 score and accuracy of the 24-feature downsampling GBDT model were both 0.83, and the area under the curve was 0.91.

**Conclusion:**

Compared with traditional models, artificial intelligence–based machine learning models have better accuracy and real-time performance and can reduce the occurrence of in-hospital MI from a data-driven perspective, thereby increasing the cure rate of patients and improving their prognosis.

## Introduction

At the beginning of 2018, the China National Center for Cardiovascular Diseases issued the Report on Cardiovascular Diseases in China 2017 (summary). The estimated number of cardiovascular disease patients in China was 290 million, and cardiovascular deaths accounted for more than 40% of all deaths, ranking first in all diseases and even higher than cancer. Cardiovascular diseases, including myocardial infarction (MI), can cause a decline in quality of life, economic difficulties, and even death [1]. One of the most lethal cardiovascular diseases, MI, is caused by unstable plaque rupture, erosion, and calcification nodules on the basis of coronary heart disease, leading to platelet aggregation and thrombosis and myocardial necrosis due to acute coronary occlusion or blocked blood flow. MI can lead to malignant arrhythmia, heart failure, and sudden death. Clinical studies have shown that early identification of and timely intervention for acute MI can significantly reduce mortality [2]. Many MI patients have a poor prognosis and even die because of weak symptoms and rapid progression.

Early coronary artery reperfusion therapy is the preferred treatment for patients with MI. The likelihood of a benefit from reperfusion therapy is negatively correlated with time [3]. Rapid identification and rescue are the keys to the success of MI treatment. Thanks to the increasing acceptance of early coronary reperfusion therapy, the acute and long-term mortality of MI has declined to some extent, but its mortality remains high. The major reason is the inability to recognize MI early, especially by noncardiovascular physicians, which delays the first medical contact and door-to-balloon time and results in poor prognosis.

The traditional MI risk assessment models are subjective, and the data that go into them are difficult to obtain. Generally, the assessment is only conducted among high-risk patient groups. Each MI patient has different causes, symptoms, and signs, but MI always progresses rapidly. The various factors make it extremely difficult for noncardiovascular physicians and even some cardiovascular physicians to accurately identify the risk of MI or monitor the risk of MI over a long time. Thus, it is impossible to formulate diagnosis and treatment plans and coordinate medical resources in advance. The MI diagnostic window for noncardiovascular physicians is longer than that for cardiovascular physicians [4], so it would be of practical significance to develop a new model that can achieve automatic identification, long-term monitoring, and timely warning of the risk of MI in each patient.

With the rapid development of computer technology and information technology, the informatization of the healthcare field has accelerated, and hospital information systems (HISs) are increasingly adopted. An HIS is a computer system for healthcare to solve problems such as medical services, patient safety, and clinical diagnosis and treatment [5, 6]. Massive amounts of data are generated by the use of an HIS system, which provides a large amount of data for real-world research.

Artificial intelligence (AI) is a technology of high research interest that can be applied to clinical diagnosis [7, 8]. Wallert et al. [9] used four popular machine learning algorithms to mine the data of 51,943 cases of new-onset MI and established a prediction model for 2-year survival after the initial onset of MI. Mansoor et al. [10] constructed an in-hospital mortality prediction model for ST-segment elevation MI in females by logistic regression (LR) and a random forest (RF) algorithm, and the area under the curve of the model reached 0.81. Researchers from the West China Hospital of Sichuan University proposed a hybrid feature selection method for the recommendation of antihypertensive drugs and combined this method with statistical analysis to screen out the key factors affecting the efficacy of antihypertensive drugs [11].

The present study aimed to develop an AI-based MI risk prediction model through machine learning applied to MI big data. We wanted a model that could be used to continuously and actively monitor all inpatients, especially noncardiovascular inpatients, thereby achieving early screening and real-time warning of MI. The model we developed can contribute to the integration, standardization, and coordination of the diagnosis and treatment process of patients at high risk for MI, reduce the occurrence of in-hospital MI through a data-driven approach, and improve the cure rate and prognosis of patients.

## Methods

### Data organization rules

The West China Hospital of Sichuan University is a large, comprehensive hospital with medical teaching and research capabilities. After receiving ethical approval, we collected data from all hospitalized patients in the West China Hospital of Sichuan University for a total of 10 years from 1 January 2011 to 31 December 2020. The inclusion criterion for the MI group was that the first page of the patient's medical record contained the keyword “myocardial infarction”. All MI patients were included in the MI group, and all non-MI patients were included in the control group. The patient data included basic patient information, electronic medical records, and laboratory test results. The basic information included admission ID number, sex, and age. The electronic medical record information included treatment department, discharge diagnosis, admission time, and discharge time. The laboratory test information included a total of 1357 laboratory test indices, such as sodium, potassium, chlorine, creatine kinase isoenzyme MB, myoglobin, creatinine, carbon dioxide binding capacity, serum β-hydroxybutyric acid, troponin-I, and troponin-T. If a patient was tested for the same laboratory test multiple times, the results of the first test were taken since the characteristics of MI patients at admission are the most obvious. Then, the basic information and electronic medical record information were combined into a complete dataset identified by the admission ID number.

### Data cleaning

According to the inclusion criteria, 20,072 patients were initially included in the MI group and 1,882,996 in the control group. The treatment of missing information was a key factor in the overall data quality [12]. Hence, we attempted to ensure the least missing information while including the most patient features. Specifically, 57 laboratory indices were selected as the features, and age and sex were taken as complimentary features, resulting in a total of 59 features. The data were preprocessed via normalization, null value deletion, nonstandard value correction, and unit conversion. Finally, the MI group included a total of 14,446 patients. The control group initially included 1,882,996 patients, which after data cleaning fell to 220,369. The feature values of all MI-positive and MI-negative patients were intact. The basic features of the MI group and control group are given in Table 1.

**Table 1:** Basic features of patients with and without MI

Feature	With MI (n = 14,446)	Without MI (n = 220,369)
Mean age (yrs)	65.9 ± 13.4	62.4 ± 16.8
Male, n (%)	11,406 (79)	131,220 (60)
Troponin-T (ng/L)	327.9 (23.4–2463.5)	12.3 (7.4–25.2)
Urodilatin (pg/ml)	1138 (348–3693.5)	242 (79–1057)
Myoglobin (ng/ml)	52.9 (29.3–183.9)	32.9 (21–64.7)
Total cholesterol (mmol/L)	3.8 (3.1–4.6)	3.94 (3.21–4.73)
Creatine kinase Isoenzymes-MB (ng/ml)	3.9 (1.9–39.3)	1.55 (1.01–2.53)
Serum creatinine (umol/L)	85.8(71–109)	74(60–93)
Fasting plasma glucose (mmol/L)	6.73(5.5–9)	5.6(4.9–7.2)
Direct bilirubin (umol/L)	4.3(3.1–6.1)	3.9(2.7–5.8)

### Machine learning model

Machine learning is a branch of AI. In machine learning, mathematical optimization is very important, including numerical calculation of system parameters [13]. This study adopted procedure-oriented programming through Python 3.6 and took target determination, problem diagnosis, program design, program execution, and evaluation iteration as machine learning training strategies. The computer CPU was an Intel Core i7-10870H 2.2GHZ, and the memory was 32 GB. Since MI risk prediction is a labelled classification problem, an MI prediction model was constructed through supervised machine learning in this study [14]. Due to the use of big data, the commonly existing problem of data imbalance needs to be addressed [15]. Therefore, the model needs to be evaluated from multiple aspects. Generally, machine learning model training is optimized by modifying the algorithm and the iteration of hyperparameters. However, considering the imbalance between the positive and negative samples in this study, the dataset construction was incorporated as a key adjustment in the model optimization.

**Fig. 1:**
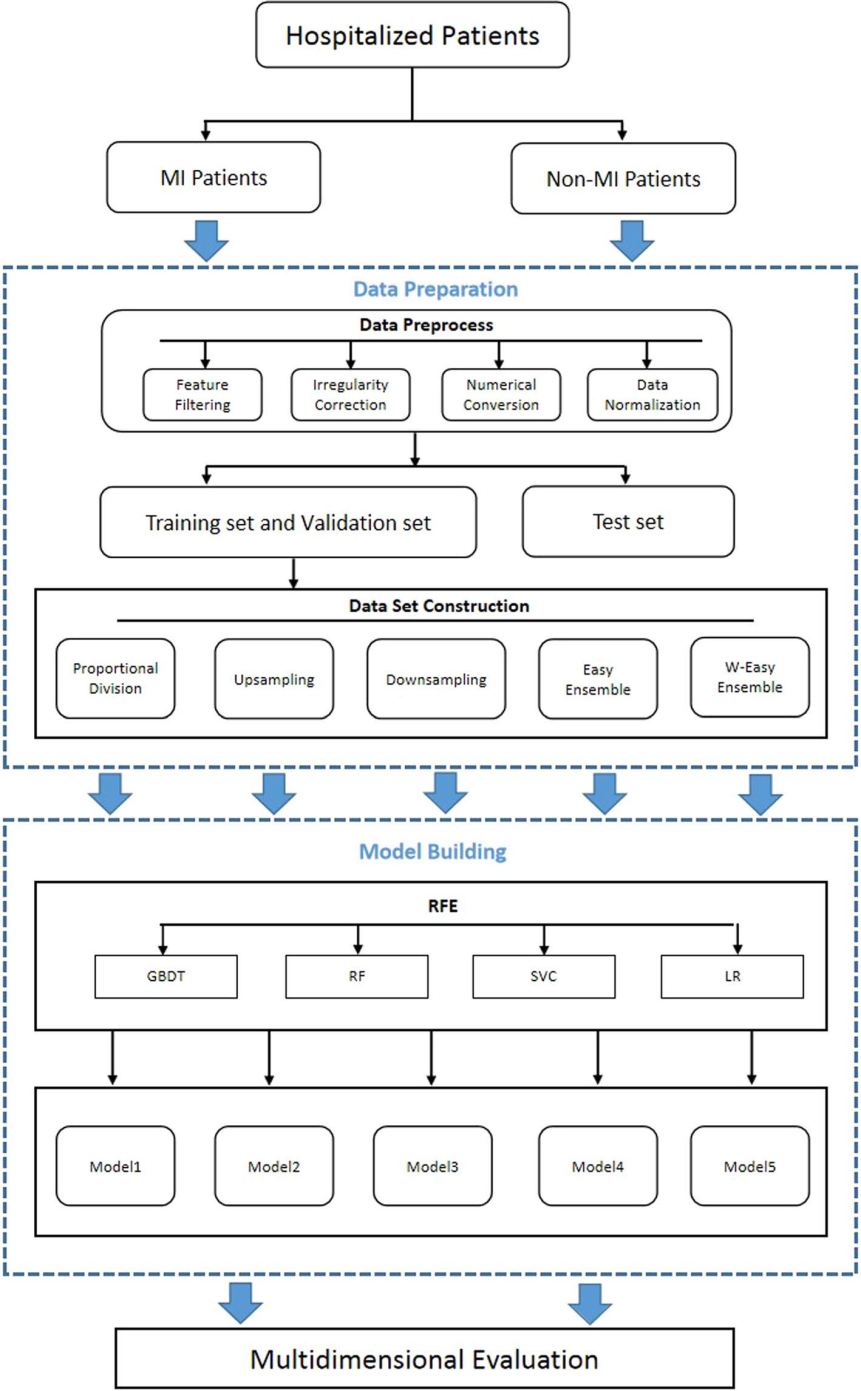
Machine learning model building flowchart

First, 1000 MI patients and 1000 control patients were randomly selected from the original dataset to verify the predictive ability and generalization ability of the model. Five datasets were constructed from the remaining data using five dataset construction methods, namely, proportional division, upsampling, downsampling, easy ensemble, and w-easy ensemble. The data in each dataset were randomly divided into a training set and a validation set at a ratio of 8:2. Finally, supervised learning was applied to train the model on each training set. Specifically, recursive feature elimination (RFE) was used as the top-layer algorithm, and RF, gradient boosting decision tree (GBDT), LR, and support vector machine were used as the bottom-layer algorithms. As an index to measure the accuracy of the binary classification model in machine learning, the F1 score takes into account the precision and recall of the classification model. Compared with the accuracy, the F1 score can better reflect the real predictive ability of the model more objectively and accurately [16]. Thus, the F1 score was used to evaluate the five models to determine the best model for MI risk prediction. The machine learning model building flow chart is shown in Fig. 1.

#### RFE

An RFE model identifies the most or least important feature, removes it from the feature set, and then repeats this on the remaining feature set until all features are traversed [17]. Finally, the feature ranking and the best feature subset are obtained to complete the modelling. The stability of RFE depends on the bottom-layer algorithm. If the bottom-layer algorithm is stable, RFE is stable, and if the bottom-layer algorithm is not stable, RFE is not stable [11]. Because the distribution of the data was not known, four bottom-layer algorithms, i.e., support vector classification (SVC), LR, RF, and GBDT, were selected for traversal and comparison. Since there were 59 features, the modelling of each algorithm was cycled 59 times to explore the best feature subset.

#### Proportional division

Model 1 was constructed by combining proportional division with RFE. All data were trained directly by proportional division, and the training set and validation set were divided according to the same ratio of positive to negative samples. The training set and validation set remained imbalanced. The training set included a total of 186,252 patients, and the validation set included 46,563 patients. The ratio of positive to negative samples in the training set was the same as that in the validation set. The RFE method constructed 236 models on the dataset, and the model with the best performance was selected and denoted as Model 1.

#### Upsampling

Model 2 was constructed by combining upsampling with RFE. Upsampling replicates the positive samples to balance the number of positive and negative samples, and the training set and validation set both maintain data balance [18]. The training set included 347,604 patients, and the validation set included 86,901 patients. The ratio of positive to negative samples was 1:1 in the training set and validation set. The RFE method constructed 236 models on the dataset, and the model with the best performance was selected and denoted as Model 2.

#### Downsampling

Model 3 was constructed by combining downsampling with RFE. In downsampling, the negative samples are resampled to balance the number of positive and negative samples, and the training set and validation set maintain data balance [18]. The training set included a total of 21,513 patients, and the validation set included 5379 patients. The ratio of positive to negative samples in the training set and validation set was 1:1. The RFE method constructed 236 models on the dataset, and the model with the best performance was selected and denoted as Model 3.

#### Easy ensemble

**Fig. 2:**
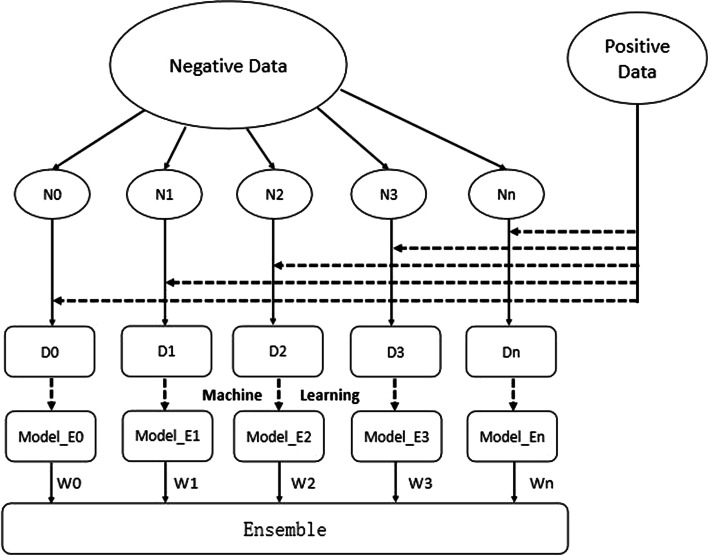
Easy ensemble, w-easy ensemble architecture diagram

Model 4 was constructed by combining the easy ensemble with RFE. The easy ensemble is based on bagging, and the final result is obtained by voting on multiple submodels [19]. As shown in Fig. 2, the easy ensemble trains all the data, including 185,823 cases in the training set and 45,993 cases in the validation set. The ratio of positive to negative samples was the same in the training set and the validation set. Unlike Model 1, the easy ensemble divided the negative samples of the training dataset into 15 subsets, each subset having the same sample number as the positive samples. Next, 15 submodels were constructed based on the 15 subsets. The submodel for each subset was the best model selected from 236 models using RFE. Then, the 15 submodels were integrated by traversing the voting difference, and the model with the best performance was selected as Model 4.

#### W-easy ensemble

Model 5 was constructed by combining the w-easy ensemble with RFE. In machine learning, weighted methods can be used to eliminate training biases [20]. W in the W-easy ensemble means weight. The W-easy ensemble adds weights to the easy ensemble method and combines bagging and boosting, as shown in Fig. 2. Each submodel of Model 4 did not have a weight, but in fact, the predictive ability of each submodel was different. To highlight the contributions of the high-quality submodels and reduce voting interference by the low-quality submodels, the F1 score was used to weight each submodel. The voting difference was traversed, and the model with the best performance was selected as Model 5.

## Results

### Results in the validation set

#### Results of Models 1–3

**Fig. 3:**
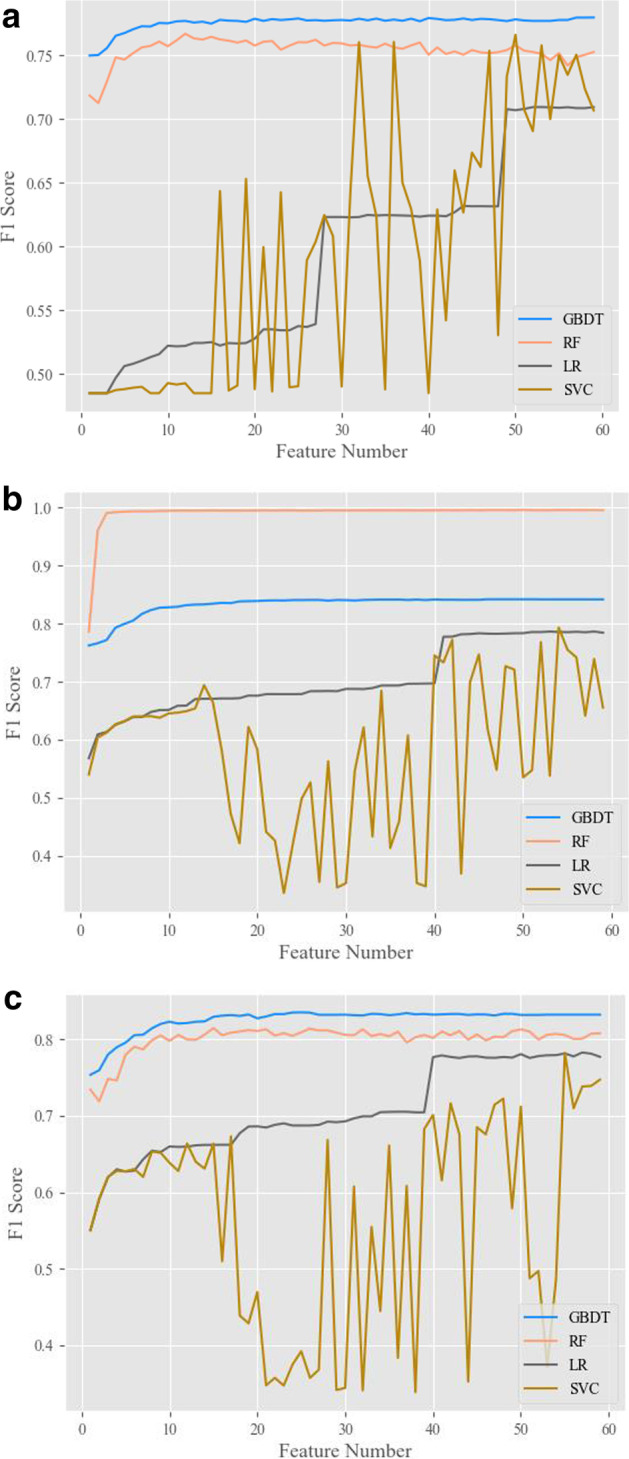
Overall results of model one, two, and three validation sets

Models 1–3 used nonensemble data construction, and the results in the validation set are shown in Fig. 3a–c. As the number of features increased, the F1 score of all models showed an overall upwards trend, yet there were large differences between different algorithms. The F1 scores of the tree-based nonlinear GBDT and RF models were significantly higher than those of the linear LR and SVC models. GBDT and RF needed fewer features than LR and SVC to reach the peak F1 score. Specifically, LR and SVC needed approximately 40 features to reach the peak F1 score, whereas GBDT and RF needed only approximately 10. In addition, the stability of GBDT and RF was significantly higher than that of LR and SVC. As shown in Fig. 3, as the number of features increased, the F1 score of LR changed in a stepwise fashion, and the F1 score of SVC changed in a zig-zag fashion. The two algorithms had large iterative differences and poor stability. Comparing GBDT and RF, it can be seen from the results of Model 1 and Model 3 that the F1 scores of GBDT were higher than those of RF in all feature combinations. Although the F1 score of RF in Model 2 reached 0.99, RF only fitted a specific dataset, which was considered overfitted [21]. Compared with GBDT, RF was more prone to overfitting. The best result of each model is given in Table 2. The downsampling GBDT model was the best model under the nonensemble data construction method. The training set had a positive sample size of 10,784 and a negative sample size of 10,729. The validation set had a positive size of 2662, a negative sample size of 2717, 24 features, and an F1 score of 0.84.

**Table 2:** Optimal results of Models 1–3 on the validation set

Model name	Construction method	Optimal algorithm	Number of optimal feature	Negative training n sample	Positive training n sample	Negative validation sample	Positive validation sample	Validation accuracy	ValidationF1 score
Model1	Proportional division	GBDT	9	175,496	10,756	43,873	2690	0.96	0.78
Model2	Upsampling	RF	3	175,395	172,209	43,974	42,927	0.99	0.99
Model3	Downsampling	GBDT	24	10,784	10,729	2662	2717	0.84	0.84

#### Results of Models 4–5

Ensemble learning is a method that combines various classifiers in a certain way to classify new instances [22]. Model 4 and Model 5 used ensemble data construction methods. The negative samples were divided into 15 subsets, and each subset included 14,625 patients, the same as the number of positive samples. Fifteen RFE submodels (Model_E0 to Model_E14) were constructed on these 15 data subsets, and the evaluation indices of each submodel were calculated independently, including the best algorithm, best feature subset, accuracy, and F1-score. As shown in Table 3, the best algorithm for each submodel was GBDT, consistent with the results of the nonensemble models. The best feature number of each submodel was approximately 20. The accuracy and F1-score were approximately 0.85, indicating strong stability.

**Table 3:** Submodel building with ensemble data

	Optimal algorithm	Optimal feature	Accuracy	F1-score
Model_E0	GBDT	18	0.87	0.87
Model_E1	GBDT	19	0.85	0.85
Model_E2	GBDT	17	0.84	0.84
Model_E3	GBDT	20	0.85	0.85
Model_E4	GBDT	20	0.84	0.84
Model_E5	GBDT	14	0.85	0.85
Model_E6	GBDT	17	0.85	0.85
Model_E7	GBDT	17	0.85	0.85
Model_E8	GBDT	14	0.85	0.85
Model_E9	GBDT	10	0.85	0.85
Model_E10	GBDT	20	0.85	0.85
Model_E11	GBDT	21	0.87	0.87
Model_E12	GBDT	16	0.89	0.89
Model_E13	GBDT	16	0.89	0.89
Model_E14	GBDT	16	0.88	0.88

The 15 submodels from Model_E0 to Model_E14 were integrated to build Model 4. Each submodel carried out prediction for the same cases, and the prediction results were combined to obtain the final result. Different combination rules produced different model results. For example, when there are more negative votes than positive votes, all predictions are negative, and when there are more positive votes than negative votes, the result of 1 vote difference being positive and 15 votes difference being positive is different. As shown in Table 4, the accuracy and F1 score gradually increased with the increase in the voting difference. When the voting difference was 15, the accuracy on the validation set reached 0.95, and the F1 score was 0.78.

**Table 4:** Voting traversal results of Model 4 and Model 5

	Vote difference	Accuracy	F1 score	Negative precision	Positive precision	Specificity	Sensitivity
Model4_1	1	0.86	0.66	0.99	0.27	0.86	0.82
Model4_3	3	0.87	0.68	0.99	0.29	0.88	0.8
Model4_5	5	0.89	0.69	0.98	0.32	0.9	0.77
Model4_7	7	0.90	0.71	0.98	0.35	0.91	0.75
Model4_9	9	0.92	0.73	0.98	0.39	0.93	0.72
Model4_11	11	0.93	0.74	0.98	0.42	0.94	0.7
Model4_13	13	0.94	0.76	0.95	0.47	0.95	0.66
Model4_15	15	0.95	0.78	0.98	0.54	0.97	0.62
Model5_0	0	0.85	0.66	0.99	0.27	0.86	0.82
Model5_1	1	0.87	0.68	0.99	0.29	0.88	0.80
Model5_2	2	0.89	0.69	0.99	0.32	0.89	0.78
Model5_3	3	0.89	0.70	0.98	0.34	0.91	0.76
Model5_4	4	0.90	0.71	0.98	0.35	0.91	0.75
Model5_5	5	0.92	0.73	0.98	0.39	0.93	0.72
Model5_6	6	0.93	0.74	0.98	0.42	0.94	0.70
Model5_7	7	0.94	0.76	0.98	0.47	0.95	0.67
Model5_8	8	0.94	0.77	0.98	0.51	0.96	0.64
Model5_9	9	0.95	0.78	0.98	0.54	0.97	0.62

In Model 5, the F1 score was introduced as the weight of each submodel of Model 4 to increase the weight of high-quality submodels and reduce the interference from low-quality models. The ensembling rules were the same as those of Model 4. Due to the introduction of weights, the range of voting differences was not [1, 15]. According to the actual data, voting differences from 0 to 9 were traversed (Table 4). For key assessment indices, the accuracy and F1 score increased synchronously with increasing voting difference, while the positive precision and sensitivity changed in opposite directions. With the threshold of 9, the accuracy on the validation set was 0.95, and the F1 score was 0.78.

The best results of Model 4 and Model 5 are shown in Table 5, and the two models showed the same performance on the validation set. A total of 45 features were required for the 15 submodels. The number of positive samples in the training set was 10,714, the number of negative samples in the training set was 175,109, the number of positive samples in the validation set was 2732, and the number of negative samples in the validation set was 43,261. The F1 score on the validation set reached 0.78, and the accuracy on the validation set reached 0.95. As in Model 1, model training and validation of Models 4 and 5 were carried out on highly imbalanced datasets, and they performed similarly to Model 1.

**Table 5:** Optimal results of Models 4 and 5 on the validation set

Model name	Construction method	Optimal Vote difference	Number of optimal feature	Negative training n sample	Positive training n sample	Negative validation n sample	Positive validation n sample	Validation accuracy	Validation F1 score
Model4	Easy ensemble	15	45	175,109	10,714	43,261	2732	0.95	0.78
Model5	W-easy ensemble	9	45	175,109	10,714	43,261	2732	0.95	0.78

### Results on the test set

**Fig. 4:**
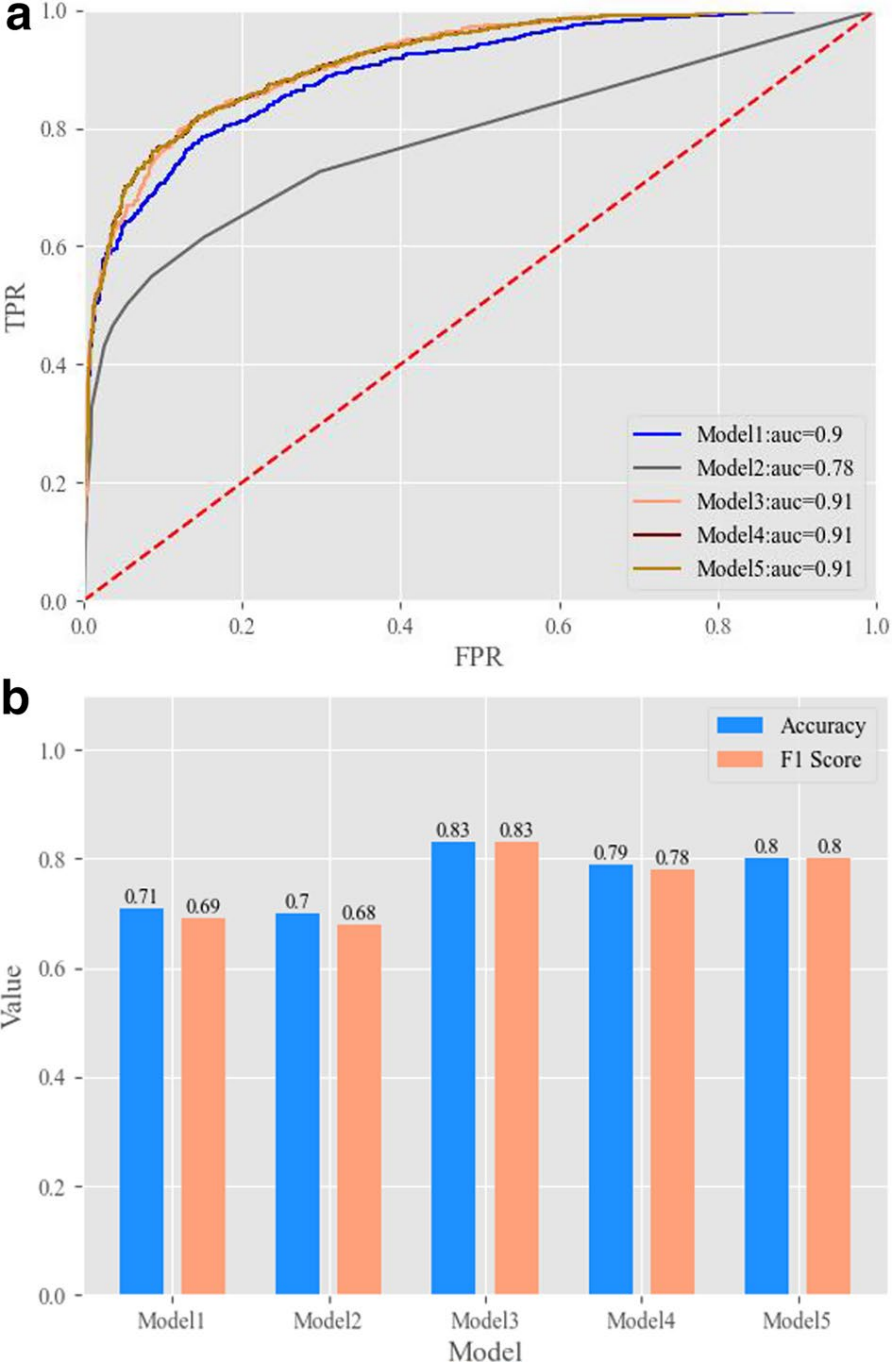
ROC curve and key evaluation indicators

The test set was not used in training or validation and was mainly used to test the generalization ability and predictive ability of the model. As shown in Table 6 and Fig. 4, the results of different models on the test set were quite different. The accuracy, F1 score, positive precision, sensitivity, negative precision, and specificity of Model 3 were all greater than 0.8, suggesting high model stability. The area under the receiver operating characteristic curve reached 0.91, indicating strong generalization ability. Based on the practical application of the model, it was difficult to completely collect all patient features. Thus, the number of features was reduced to 15 at the expense of 0.01 accuracy and the F1 score to meet real-world needs. The 15 features were key features indicating whether a patient had MI (Table 7). Some of these features have been clinically verified as key features. For example, troponin-T and creatine kinase isoenzyme-MB are sensitive indices of acute MI [23, 24]. Creatinine might also have certain predictive value for the occurrence of cardiovascular diseases [25]. Features such as urodilatin, total cholesterol, and monocyte percentage, which have not been clinically verified, are potential indices obtained through data-driven mining and can provide references for clinical explorations and research into MI prediction.

**Table 6:** Results of all models on the test set

	Accuracy	F1 score	Negative precision	Positive precision	Specificity	Sensitivity	AUC
Model1	0.71	0.69	0.64	0.98	0.99	0.44	0.90
Model2	0.70	0.68	0.63	0.94	0.97	0.42	0.78
Model3	0.83	0.83	0.81	0.87	0.88	0.80	0.91
Model4	0.79	0.78	0.71	0.94	0.96	0.61	0.91
Model5	0.80	0.80	0.73	0.93	0.95	0.65	0.91

**Table 7:** Key features used for MI risk prediction

Feature name	Description	Normal reference (unit)
TNT	Troponin-T	0–14 ng/L
UD	Urodilatin	0–227 pg/ml
MB	Myoglobin	20–80 ng/ml
ALB	Albumin	35–50 g/L
TC	Total cholesterol	2.9–6 mmol/L
Cl	Plasma chlorine	96–106 mmol/L
CK-MB	creatine kinase isoenzymes-MB	0–4.94 ng/ml
Cr	Serum creatinine	54–106 umol/L
MONO%	Monocyte percent	3–8%
FPG	Fasting plasma glucose	3.9–6.1 mmol/L
DBil	Direct bilirubin	0–6.8 umol/L
AG	Anion gap	8–16 mmol/L
IBil	Indirect bilirubin	1.7–10.2 umol/L
Age		
Sex		

## Discussion

With the rapid growth of big medical data, emerging technologies such as AI and machine learning have been increasingly applied in the medical field. [26, 27]. Due to the use of big data, data imbalance is a common problem. Data imbalance refers to the imbalance of the ratio of positive to negative samples in the actual dataset. Extreme imbalance is a characteristic feature of medical big data [28]. Out of the total population, people with a given disease account for only a small portion. Traditional machine learning models are mostly trained using balanced datasets, which makes it difficult to directly apply imbalanced datasets. Therefore, in this study, from the perspective of different dataset construction methods, we constructed multiple models to deal with data imbalances, evaluated the differences between different models from multiple aspects, analysed the underlying causes of the differences, and ultimately constructed an MI risk prediction model for practical implementation.

In Model 1, all data were proportionally divided into the training and validation sets. The training data and validation data were both imbalanced, which is consistent with the real-world situations. Model 1 performed poorly on both the validation set and the test set. This was because in the training process, the optimization objective was accuracy. For such extremely imbalanced data, in extreme cases in which all predictions are negative, the accuracy can exceed 95%. Therefore, the model is more inclined to negative prediction to maximize the overall accuracy while reducing the model complexity, but in fact, it did not learn the data distribution characteristics.

Model 2 included all the data for training and verification, and the positive data were replicated to match the number of negative samples. The performance of Model 2 on the validation set is really good (F1-score = 0.99), whereas decreases significantly in the test set (F1-score = 0.68), suggesting that it might be due to overfitting. Due to the repetition of positive data in the training and verification sets, model 2 actually only fitted the training set, and the results of the validation set were distorted.

Model 3 downsampled the negative data randomly to balance the data in both the training dataset and the validation dataset, which avoided the learning bias of Model 1 and the repeated training of Model 2. This made it easier for Model 3 to learn the characteristic distribution of the data. Although the results on the validation set and the test set were good, unlike Model 1 or Model 2, not all the data were used in Model 3. The negative dataset of Model 3 was generated by random sampling, which brings errors and uncertainties. The results of different sampling methods were often different, resulting in poor stability of the model.

To improve the stability of Model 3, Model 4 adopted ensemble learning based on bagging and used all the data for training and validation, which effectively reduced the prediction variance [29]. The final result was obtained by building multiple submodels. For the same case, if most submodels predicted positive data, the likelihood that the data were positive was high. Compared with a single submodel, such as Model 3, Model 4 had higher stability because all data were used for its training. Although Model 1, Model 2, and Model 4 all used all the data for training, Model 4 had significantly better results on the test set compared with Model 1 and Model 2, indicating that Model 4 had learned the distribution of the MI characteristics by reading the full dataset and had no learning bias or overfitting. Moreover, different results could be obtained by controlling the voting difference to meet the needs of different application scenarios. It can be seen from Table 4 that with the increase in the voting difference, the confidence level of the positive prediction grew increasingly higher, and the accuracy of the positive prediction was also increasingly higher, reflecting that the positive prediction of the model became increasingly cautious. In clinical practice, this is called a low misdiagnosis rate. However, sensitivity has become increasingly lower, which is called a high missed diagnosis rate in clinical practice. Therefore, many patients with MI are considered non-MI patients. Unlike Model 3, Model 4 was not affected by sampling error, so it was more stable and flexible. However, due to the different key features of different submodels, the number of parallel features required by the ensemble model reached 45. Thus, it is impractical to use such a model in medical practice. Moreover, different submodels had different prediction capabilities and were prone to producing noise interference. For these reasons, the results of Model 4 on the validation set and test set were of medium quality.

Model 5 solved the noise problem of Model 4 based on a hybrid ensemble learning idea of bagging plus boosting. Bagging submodels were generated in parallel with no weights, and boosting submodels were generated in serial with weights. Therefore, the boosting weights were introduced to bagging submodels, in which the weights were the F1 scores of the submodels. The higher the F1 score of the high-quality model is, the greater the weight is, while the lower the F1 score of the noisy model is, the smaller the weight is. Hence, reasonable results were obtained. Moreover, as in Model 4, the voting difference could be traversed to observe the changes in the accuracy and sensitivity, and thereby, a reasonable model could be flexibly selected. The performance of Model 5 on the validation set was similar to that of Model 4, but the results on the test set were slightly better than those of Model 4, suggesting stronger generalization ability and stability.

The bottom-layer algorithm of each model is shown in Fig. 3a–c. Nonlinear models such as GBDT and RF were superior to linear models such as LR and SVC. GBDT and RF have fewer features, higher accuracy, and higher F1 scores. Therefore, nonlinear models may be more suitable for datasets with a high data dimension, a large data volume, and a difficult-to-find best linear segmentation hyperplane. The GBDT is generated based on boosting, and the RF is generated based on bagging. GBDT yielded better model results than RF, yet the training efficiency of RF was higher. On the same dataset, the training speed of RF was approximately 10 times that of GBDT. In this study, we chose GBDT as the bottom-layer algorithm and RFE as the top-layer algorithm for optimal feature subset traversal.

Generally, when evaluating a supervised machine learning model, most of them are evaluated in a balanced dataset, which are likely to deviate from the real world. For example, Model 3 had high accuracy and a high F1 score (0.83) on the test set, yet it did not perform well on the Model 5 validation set with extremely imbalanced positive and negative data, having an F1 score of only 0.67. This is also the reason why some AI models do not meet clinical expectations in real-world implementation.

In such situations, it is necessary to have correct expectations and understanding of the evaluation index. A value of 0.67 does not mean that it is an invalid model. AI models pay more attention to the accuracy and sensitivity, corresponding to clinical misdiagnosis and missed diagnosis. In the real world, most AI models cannot achieve a low misdiagnosis rate or a low missed diagnosis rate at the same time. A low misdiagnosis rate of positive cases indicates that the diagnosis is cautious, which will lead to missed diagnosis. In contrast, a low missed diagnosis rate of positive cases indicates a rough diagnosis and may raise the misdiagnosis rate. Of these two types of errors, clinical practice would prefer misdiagnosis over missed diagnosis, i.e., a low missed diagnosis rate.

Although the F1 score of Model 3 in the imbalanced dataset is only 0.67, its sensitivity is 0.83, specificity is 0.87, positive accuracy rate is 0.3, and negative accuracy rate is 0.99. Therefore, although not all positive patients predicted by the model will have MI, and maybe only a small portion of them will, the model essentially helps identify high-risk patients who need to receive early intervention and close observation. The model is suitable for clinical application, so it has high practical application value.

Similarly, the change in the voting difference for Model 5 can achieve dynamic adjustment of the misdiagnosis rate and the missed diagnosis rate. For example, in the case of a voting difference of 0, the missed diagnoses of positive cases were only 18%, which was more consistent with the current clinical scenarios than the 38% missed diagnosis of positive cases when the voting difference was 9. Moreover, the accuracy and F1 score of the model with a voting difference of 0 on the test set were both 0.84, even higher than those of Model 3. Therefore, the model with a voting difference of 0 is better than the model with a voting difference of 9. It is inadequate to evaluate the indices with the accuracy and F1 score alone in some cases. It is necessary to understand the connotation of the indices, the data distribution, and the characteristics of the application scenario to select the best model. Considering all these factors, Model 3 was chosen as the best model for MI risk prediction in this study.

Some common clinical MI risk assessment methods, such as the ITF/IAS guidelines, have set a threshold of only 32% predictive power for positive results [30]. These scale models can only achieve medium accuracy and have shortcomings such as a lack of adjustment parameters, few features, and lack of timeliness. Moreover, the data of the scale models are subjective and difficult to obtain. Generally, the scale models are only for evaluating high-risk patients and are not applicable to other patients in the whole hospital. Since nonhigh-risk patients are not monitored, once MI occurs, it is very easy for a poor prognosis and even death to result due to untimely rescue and incorrect treatment methods. Currently, for the prevention and treatment of MI, hospitals in China generally do not have a hospital-wide coordination system for the early identification or early warning of MI. Although the construction of chest pain centers in hospitals has optimized the treatment process of acute MI in hospitals, there is no corresponding HIS to support the operation of the model, so the rescue ability is low.

Therefore, AI-based risk prediction models for MI will have great value. With real-world data, this study used machine learning to mine big data on MI. In the process, data imbalance was taken into account, and multiple models were constructed for directional optimization. Finally, the MI risk prediction model was applied to the HIS system to monitor the risk of MI in all hospitalized patients in real time, thereby achieving automatic early warning. The model provided strong information support for the construction of regional MI prevention and management systems. As a result, noncardiovascular physicians could pay more attention to the risk of MI in patients, and cardiovascular physicians could have more reference data. Moreover, the proposed model could provide decision-making support for primary care physicians to better grasp the characteristics of disease changes and formulate reasonable treatment plans, which will also promote the optimization of medical resource allocation and the implementation of hierarchical diagnosis and treatment systems.

## Data Availability

The datasets generated and/or analysed during the current study are not publicly available due to local regulations on the management of medical records.
